# In-Hospital and Long-Term Outcomes in Patients with Head and Neck Cancer Bleeding

**DOI:** 10.3390/medicina58020177

**Published:** 2022-01-25

**Authors:** Chieh-Ching Yen, Che-Fang Ho, Chia-Chien Wu, Yu-Ning Tsao, Chung-Hsien Chaou, Shou-Yen Chen, Chip-Jin Ng, Heng Yeh

**Affiliations:** 1 Department of Emergency Medicine, Chang Gung Memorial Hospital, Linkou Branch, Taoyuan 33305, Taiwan; chiehching74@gmail.com (C.-C.Y.); shien@url.com.tw (C.-H.C.); allendream0621@yahoo.com.tw (S.-Y.C.); ngowl@ms3.hinet.net (C.-J.N.); 2 College of Medicine, National Yang Ming University, Taipei 11221, Taiwan; 3 Department of Otolaryngology Head and Neck Surgery, Chang Gung Memorial Hospital, Keelung 20401, Taiwan; justho@cgmh.org.tw; 4 Department of Medical Imaging and Intervention, Chang Gung Memorial Hospital, Linkou Branch, Taoyuan 33305, Taiwan; gachine105@hotmail.com; 5 Department of Otolaryngology Head and Neck Surgery, Chang Gung Memorial Hospital, Linkou Branch, Taoyuan 33305, Taiwan; yuning526210@hotmail.com; 6 College of Medicine, Chang Gung University, Taoyuan 33302, Taiwan; 7 Chang Gung Medical Education Research Center, Taoyuan 33305, Taiwan

**Keywords:** head and neck cancer, bleeding, hemorrhage

## Abstract

*Background and Objectives*: The purpose of the present study was to elucidate the in-hospital and long-term outcomes of patients with head and neck cancer (HNC) bleeding and to analyze the risk factors for mortality. *Materials and Methods*: We included patients who presented to the emergency department (ED) with HNC bleeding. Variables of patients who survived and died were compared and associated factors were investigated by logistic regression and Cox’s proportional hazard model. *Results*: A total of 125 patients were enrolled in the present study. Fifty-nine (52.8%) patients experienced a recurrent bleeding event. The in-hospital mortality rate was 16%. The overall survival at 1, 3 and 5 years was 48%, 41% and 34%, respectively. The median survival time was 9.2 months. Multivariate logistic regression analyses revealed that risk factors for in-hospital mortality were inotropic support (OR = 10.41; Cl 1.81–59.84; *p* = 0.009), hypopharyngeal cancer (OR = 4.32; Cl 1.29–14.46; *p* = 0.018), and M stage (OR = 5.90; Cl 1.07–32.70; *p* = 0.042). Multivariate Cox regression analyses indicate that heart rate >110 (beats/min) (HR = 2.02; Cl 1.16–3.51; *p* = 0.013), inotropic support (HR = 3.25; Cl 1.20–8.82; *p* = 0.021), and hypopharygneal cancer (HR = 2.22; Cl 1.21–4.06; *p* = 0.010) were all significant independent predictors of poorer overall survival. *Conclusions*: HNC bleeding commonly represents the advanced disease stage. Recognition of associated factors aids in the risk stratification of patients with HNC bleeding.

## 1. Introduction

Head and neck cancer (HNC) comprises various histopathological tumors, mostly squamous cell carcinoma [1], and it occurs within the head and neck area, including the oral cavity, nasal cavity, paranasal sinuses, oropharynx, larynx, and nasopharynx [2]. Approximately 700,000 people worldwide are newly diagnosed with HNC each year, while 380,000 worldwide die of HNC, including more than 10,000 in the United States alone [3]. In Taiwan, given the unique lifestyle factor of widespread betel nut chewing, the incidence of HNC has increased, and the age of the patients has decreased. In 2016, the incidence rate of HNC was 33.1 per 10,000 with a mortality rate of 12.5 per 10,000 among the general population and approximately 2936 deaths [4,5].

The estimated incidence of hemorrhage in cancer patients is 6–14%, and HNCs are the most common cause of intractable hemorrhage with an incidence rate ranging from 0.5–10% [4]. When life-threatening bleeding occurs, initial resuscitation and timely standardized treatment by multidisciplinary teams are important for quick identification and control of the source of bleeding. Traditionally, bleeding is managed by surgical intervention with either repair or ligation of the artery [6]. In recent years, radiological interventions, such as deconstruction using endovascular embolization or reconstruction with covered stents, have become first-line treatments for HNC bleeding if there are contrast media extravasation, pseudoaneurysm, and irregular artery contours according to angiography findings [6,7,8].

Despite the improved treatment modalities, the outcomes of patients with HNC bleeding remain dismal due to acute clinical severity and advanced cancer stage [9,10,11,12,13]. However, few studies have reported prognostic factors of short-term and long-term outcomes in patients with HNC bleeding. The aim of the present study was to examine the risk factors for in-hospital mortality and predictors of long-term overall survival in patients with HNC bleeding.

## 2. Materials and Methods

### 2.1. Study Design and Setting

The present study was approved by the Chang Gung Medical Foundation Institutional Review Board (IRB no. 202101362B0) and was performed in accordance with the Declaration of Helsinki. All adult patients who met the inclusion criteria in the study from 1 January 2015 to 31 December 2016 were retrospectively enrolled for analysis. The present study was performed at a tertiary referral center that had a capacity of 3700 beds, 100,000 annual admissions, and 200,000 emergency department (ED) annual visits in Taiwan.

### 2.2. Patient Selection and Data Collection

Through a computerized search of the electronic medical records (EMRs) during the study period, we first identified all adult patients with the International Classification of Diseases (ICD)-10 codes, C00–C14 and C30–C32, of head and neck cancer (HNC) who were treated at the ED. Second, we determined eligible patients using the following keywords: bleeding, hemorrhage, and carotid blowout. We excluded patients with incomplete medical records, duplicate ED visit data, and bleeding events originating from other sites, such as gastrointestinal bleeding, intracranial hemorrhage, and vaginal bleeding. The patients selected by EMRs were reviewed by two physicians (H.-Y. and Y.-N.T.).

Demographic information, such as age, sex, lifestyle factors, initial ED vital signs, and previous medical history, including hypertension, diabetes mellitus, coronary artery disease, chronic kidney disease, other malignancy, prior stroke, and liver cirrhosis, was collected. Laboratory findings on initial presentation included white cell count, hemoglobin, platelet count, international normalized ratio, creatinine, and alanine aminotransferase. Information of the presentation of primary cancer, including cancer site, pathologic type, initial cancer treatment modality, local recurrence, tumor-node-metastasis (TNM) staging system at the time of HNC bleeding according to the American Joint Committee on Cancer 7th edition, was obtained.

Computed tomography (CT) angiography was performed in patients with active life-threatening HNC bleeding if treatment with local compression and packing failed. Mechanisms of HNC bleeding consisted of tumor-related causes, pseudoaneurysm, and post-operative complications. Tumor related causes were defined as contrast extravasation, hypervascular tumor staining, or great vessel involvement on CT angiography, or bleeding from a necrotic wound of the tumor confirmed by otolaryngologists with or without a fiberoptic endoscopy. Pseudoaneurysm was all confirmed by CT angiography.

Patients with HNC bleeding received treatment involving supportive care, endovascular therapy, and surgical intervention. Supportive care was defined as medication with oral or intravenous tranexamic acid, epinephrine-soaked gauze compression and packing, or observation. Endovascular therapy consisted of transarterial embolization and covered stent graft placement, while surgical intervention consisted of surgical ligation and primary repair. Patients who required blood transfusion and inotropic support were also described.

The primary outcomes were in-hospital mortality and overall survival, and the secondary outcome was the recurrent bleeding incidence rate after the initial bleeding presentation. Patients were followed up from the date of diagnosis with HNC bleeding until death or June 2021.

### 2.3. Statistical Analysis

Patient characteristics, previous medical history, laboratory findings, and presentations of cancer and bleeding were reported as numbers (percentages) for categorical variables and mean ± standard deviation (SD) for continuous variables. Comparisons between survivors and non-survivors were made by the chi-square test or Fisher’s exact test as appropriate for categorical variables. Independent Student’s *t*-tests were used for normally distributed continuous variables, and Mann–Whitney U-tests were used for skewed continuous variables. Multivariable logistic regression models that included variables associated with a higher risk of in-hospital mortality at a *p* < 0.1 in univariate analysis were conducted to evaluate the impact of those variables on in-hospital mortality. To identify independent predictors of long-term overall survival, we used a stepwise approach to select variables with *p* < 0.1 in univariate analysis to enter the final multivariate Cox proportional hazards model. Cumulative survival rates were depicted by Kaplan–Meier curves and compared by log-rank tests for each variable, which was determined to be significant in the multivariate analysis. All analyses were performed using SPSS software v26 (SPSS Inc., Chicago, IL, USA). A two-sided *p* value of <0.05 was considered statistically significant.

## 3. Results

### 3.1. Patient Characteristics between Survivors and Non-Survivors

**Figure 1:**
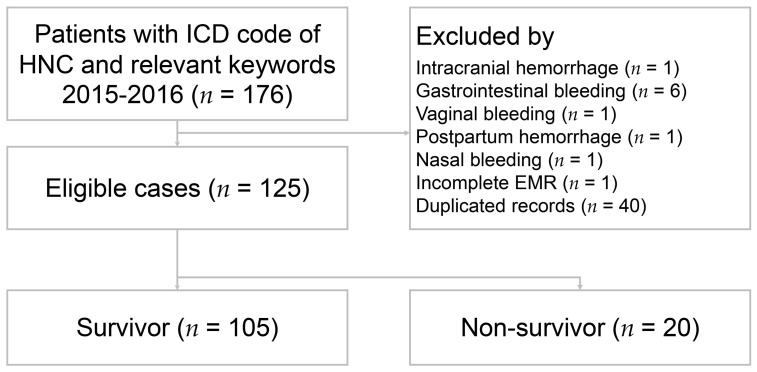
Flow chart of patient selection. ICD: International Classification of Diseases; HNC: head and neck cancer; EMR: electronic medical records.

A total of 125 patients met the entry criteria for the study (Figure 1). The patient characteristics are presented in Table 1. Of the 125 patients, 76 (60.8%) were hospitalized, 45 (36%) were discharged from the ED, and 4 (3.2%) died in the ED. Males accounted for the majority of patients (*n* = 114, 91.2%), and the mean age was 55.9 ± 11.1 years. The distributions of age and sex did not differ between survivors and non-survivors. The number of patients who survived hospitalization was 105 (84%), and the number who did not survive hospitalization was 20 (16%). Of all patients with HNC bleeding, the oral cavity was the most common site (*n* = 55, 44%) followed by the oropharynx (*n* = 24, 19.2%), hypopharynx (*n* = 22, 17.6%), nasopharynx (*n* = 20, 16%), and larynx (*n* = 4, 3.2%). More than half of patients had the advanced HNC stage, and almost all patients had pathologically confirmed squamous cell carcinoma (*n* = 124, 99.2%). Fifty-eight (46.4%) patients had local recurrence. With respect to initial cancer treatment, 59 (47.2%) patients received surgical resection. Among them, 52 had concomitant neck dissection, and 43 had concurrent flap reconstructions. Moreover, 100 (80%) patients underwent adjuvant concurrent chemoradiotherapy, and 15 (12%) patients had no cancer-associated treatment either due to perceived concerns about treatment side effects or therapy not yet having been arranged at the time of diagnosis (Table 2).

### 3.2. Bleeding and Various Treatment Modalities

Extraoral bleeding accounted for the predominant site of bleeding origin (*n* = 89, 71.2%) among all HNC patients. More than one-half of the patients (*n* = 81, 64.8%) presented with active bleeding, while the remainder (*n* = 44, 35.2%) had self-limited bleeding. Nearly one-half of the patients (*n* = 62, 49.6%) required emergent computed tomography (CT) angiography for bleeder localization and further management. Among them, 4 (6.5%) patients had contrast extravasation on imaging (three from the external carotid artery and one from the internal carotid artery), 16 (25.8%) had pseudoaneurysms (nine from the external carotid artery, five from the internal carotid artery, and two from the common carotid artery), and five (8%) had pseudoaneurysms combined with contrast extravasation (three from the external carotid artery, one from the internal carotid artery, and one from the common carotid artery). The mechanisms of HNC bleeding were as follows: tumor related (*n* = 96, 76.8%); pseudoaneurysm (*n* = 21, 16.8%); and postoperative complications (*n* = 8, 6.4%). With regard to bleeding management, most patients (*n* = 93, 74.4%) received only supportive care, but other treatments included transarterial embolization (*n* = 23, 18.4%), covered stent placement (*n* = 5, 4%), surgical ligation (*n* = 3, 2.4%), and primary repair (*n* = 1, 0.8%). Fifteen (12%) patients, all in the survivor group, received further hemostatic radiotherapy. The mortality rates of supportive care and transarterial embolization were 17.2% (16/93) and 17.4% (4/23), respectively. Patients who underwent covered stent placement, surgical ligation, and primary repair all survived to hospital discharge. Given the study design and small number of patients, we did not compare the various treatment modalities for outcome assessment. In the acute setting, nearly one-half of patients (*n* = 65, 52%) underwent blood transfusion, while seven (5.6%) patients required inotropic support owing to hemodynamic instability. The non-survivor group showed a significantly higher rate of inotropic support than the survivor group (20 vs. 2.9%, *p* = 0.013) (Table 2).

### 3.3. Univariate and Multivariate Logistic Regression Analyses of in-Hospital Mortality

In total, 29 (16%) patients died during hospitalization, and among these patients, 11 (55%) died of tumor rebleeding, seven (35%) died of aspiration pneumonia, one (5%) died of tumor-related airway obstruction, and one (5%) died of sepsis. Univariate and multivariate logistic regression analyses were employed to assess risk factors for in-hospital mortality. Univariate risk factors included inotropic support (OR = 8.50; Cl 1.74–41.56; *p* = 0.008) and hypopharyngeal cancer (OR = 4.33; Cl 1.51–12.47; *p* = 0.007). After adjustment, multivariate risk factors for in-hospital mortality were inotropic support (OR = 10.41; Cl 1.81–59.84; *p* = 0.009), hypopharyngeal cancer (OR = 4.32; Cl 1.29–14.46; *p* = 0.018), and M stage (OR = 5.90; Cl 1.07–32.70; *p* = 0.042) (Table 3).

### 3.4. Long-Term Mortality and Survival Analysis for Patients with Head and Neck Cancer (HNC) Bleeding

**Figure 2:**
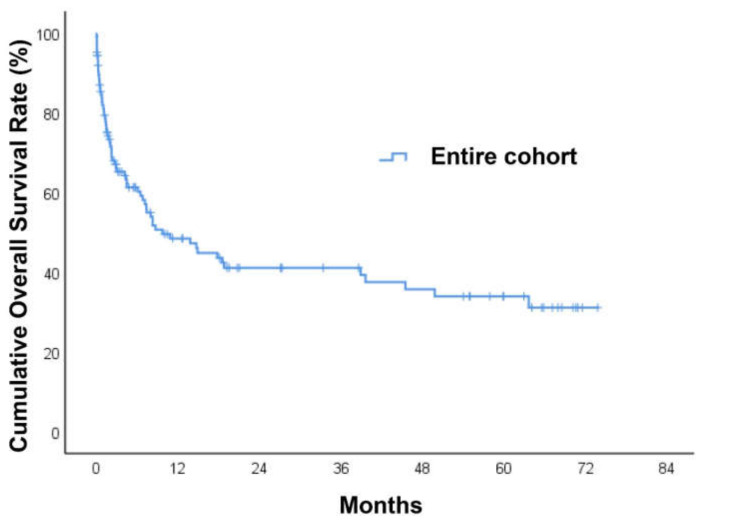
Kaplan–Meier survival curves of patients with head and neck cancer bleeding.

**Figure 3:**
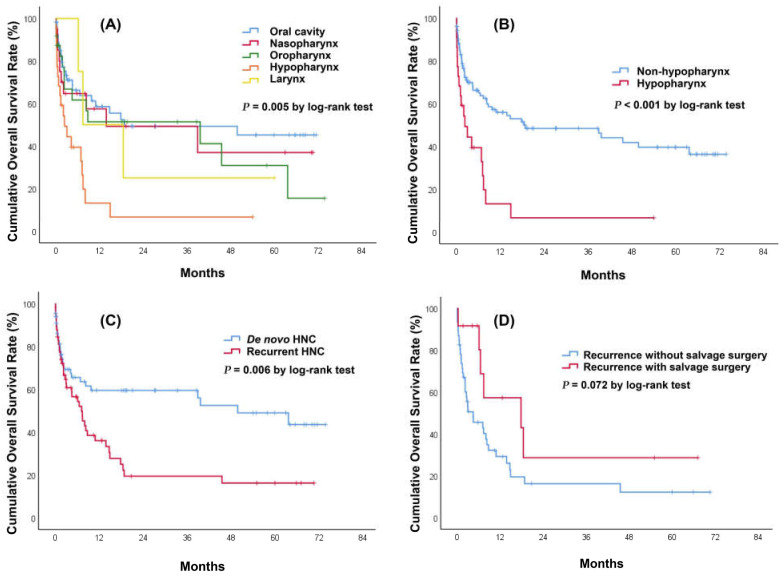
Kaplan–Meier survival curves of patients with head and neck cancer bleeding. (**A**) Stratified by cancer sites. There were significant differences in survival time between hypopharyngeal cancer and oropharyngeal cancer (*p* = 0.010), hypopharyngeal cancer and nasopharyngeal cancer (*p* = 0.016), and hypopharyngeal cancer and oral cavity cancer (*p* < 0.001). There was no significant difference in survival time between oral cavity cancer and nasopharyngeal cancer (*p* = 0.626), oral cavity cancer and oropharyngeal cancer (*p* = 0.373), oral cavity cancer and laryngeal cancer (*p* = 0.690), nasopharyngeal cancer and oropharyngeal cancer (*p* = 0.846), nasopharyngeal cancer and laryngeal cancer (*p* = 0.853), oropharyngeal cancer and laryngeal cancer (*p* = 0.887), and hypopharyngeal cancer and laryngeal cancer (*p* = 0.110). (**B**) Stratified by patients with or without hypopharyngeal cancer. (**C**) Stratified by patients with de novo or recurrent HNC (*p* = 0.006). (**D**) Stratified by patients with or without salvage surgery among recurrent HNC patients (*p* = 0.072).

During a median follow-up period of 4.5 months (IQR: 1.1–19.3) after a diagnosis of HNC bleeding, 68 of 125 patients (54.4%) died. Among the entire cohort, the 30-day mortality rate after patients developed HNC bleeding was 19%. The overall survival at 1, 3, and 5 years was 48%, 41%, and 34%, respectively. The median survival time was 9.2 months (Figure 2). When stratifying the survival time by HNC site, we found that hypopharyngeal cancer had the lowest 1-year overall survival (13%) followed by laryngeal cancer (50%), oropharyngeal cancer (51%), nasopharyngeal cancer (57%), and oral cavity cancer (59%) (*p* = 0.005) (Figure 3A). When patients were divided into a hypopharyngeal cancer group and a non-hypopharyngeal cancer group, Kaplan–Meier analysis showed that the former group had a significantly lower survival curve than the latter (*p* < 0.001) (Figure 3B). The patients with recurrent HNC had significantly decreased overall survival compared to those with de novo HNC (*p* = 0.006) (Figure 3C). We further performed subgroup analysis among patients with recurrent HNC. The patients who underwent salvage surgery had a non-significantly higher survival curve than those without salvage surgery (*p* = 0.072) (Figure 3D). Univariate and multivariate Cox regression analyses were used to investigate predictors of long-term overall survival. Univariate predictors included heart rate >110 (beats/min) (HR = 1.95; Cl 1.18–3.23; *p* = 0.010), inotropic support (HR = 5.71; Cl 2.40–13.57; *p* < 0.001), hypopharygneal cancer (HR = 2.79; Cl 1.60–4.85; *p* < 0.001), surgical resection (HR = 0.57; Cl 0.35–0.92; *p* = 0.023), chemoradiation (HR = 2.55; Cl 1.21–5.37; *p* = 0.014), T stage (HR = 1.40; Cl 1.10–1.78; *p* = 0.006), N stage (HR = 1.33; Cl 1.06–1.68; *p* = 0.015), and local recurrence (HR = 1.95; Cl 1.20–3.19; *p* = 0.007). Multivariate analyses indicated that heart rate >110 (beats/min) (HR = 2.02; Cl 1.16–3.51; *p* = 0.013), inotropic support (HR = 3.25; Cl 1.20–8.82; *p* = 0.021), and hypopharygneal cancer (HR = 2.22; Cl 1.21–4.06; *p* = 0.010) were all statistically significant independent predictors of poorer overall survival (Table 4).

### 3.5. Recurrent Bleeding Assessment in HNC Patients

**Figure 4:**
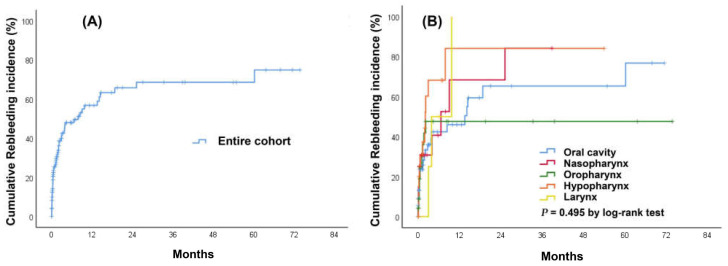
Cumulative incidence curves of rebleeding in patients with head and neck cancer. (**A**) entire cohort. (**B**) stratified by cancer sites.

In total, 59 (52.8%) patients experienced a recurrent bleeding event, and among these patients, the median time to rebleeding was 33 days (IQR: 6–112) after a diagnosis of index HNC bleeding. Moreover, 29 (49.2%) patients required emergent CT angiography for further management, and 18 (30.5%) patients died after readmission. The cumulative incidence rate of rebleeding was 25% at 30 days, 48% at 180 days, and 57% at 1 year. There were no significant differences in the incidence rate of rebleeding among various HNC sites (*p* = 0.495) (Figure 4). Univariate analyses did not identify any predictors significantly associated with an increased risk of rebleeding (not shown).

## 4. Discussion

The present study was a retrospective study that evaluated the difference between survivors and non-survivors among patients with HNC bleeding in terms of in-hospital and long-term mortality. The major findings of the present study were as follows: (1) the in-hospital mortality rate was 16% (20/125), and the long-term median survival time was 9.2 months; (2) the rate of rebleeding was 52.8%, and the median time to rebleeding was 33 days; and (3) multivariate analyses showed that inotropic support, hypopharyngeal cancer, and M stage were significant risk factors for in-hospital mortality, while heart rate >110 (beats/min), inotropic support, and hypopharygneal cancer were significant predictors of poorer long-term overall survival. To the best of our knowledge, the present study represents the largest cohort of patients with HNC bleeding.

HNC bleeding results from local vessel damage caused by direct tumor invasion, chemotherapy, local tumor irradiation, surgical procedure (e.g., radical neck dissection), post-operative non-healing wounds, the presence of a pharyngocutaneous fistula, nutritional factors, and systemic processes, such as intravascular coagulopathy or thrombocytopenia [14]. Previous studies have mostly focused on the management and outcome of carotid blowout syndrome (CBS), which is a life-threatening complication of advanced HNC [8,9,10,11,15,16,17,18,19,20,21]. CBS occurs in approximately 4% of all HNC cases and is defined as the rupture of the carotid artery or its extracranial branches, which typically involve the common carotid artery, internal carotid artery, and proximal external carotid artery [15]. However, CBS accounts for only a portion of HNC bleeding, and such studies may preclude a large majority of HNC bleeding patients. It is worth noting that 49.6% of patients with HNC bleeding who presented to the ED required emergent CT angiography, and 59.7% of those did not have contrast extravasation or pseudoaneurysm on imaging in our cohort. Cannavale et al. retrospectively reviewed patients who underwent invasive angiography for HNC bleeding and reported that 87.5% of patients were negative for active bleeding on CT angiography, while 33% of patients had no target bleeding on invasive angiography. The reasons for a low positive yield rate on imaging could be that the clinically apparent bleeding was usually self-limited and from the tumor itself rather than carotid artery involvement since there was hypervascular tumor staining on invasive angiography in some patients [22].

The mortality rate of CBS ranges from 3% to 50% in the literature [23,24]. The recent largest cohort study on CBS from Chen et al. reported a 30-day mortality rate of 21.8% in patients undergoing emergency management [20]. The results were similar to our data, in which the 30-day mortality rate was 19%, despite different disease pattern. With regard to overall survival, Lu et al. analyzed 45 patients with CBS and found that the median survival was 12 months. Liang et al. included 37 patients with CBS and demonstrated that the survival rate was 70% at 30 days and 37% at 1 year. The present study revealed poor prognosis in patients with HNC bleeding with a median overall survival of 9.2 months. We also found that more than one-half of patients died of tumor bleeding. Although these findings raise the question of whether HNC bleeding is a predictor for long-term survival, we cannot clarify this issue given that there was no control group in our study. Further large-scale studies with a control group are needed to confirm our findings.

In the present study, we found that hypopharyngeal cancer was an independent risk factor for in-hospital mortality and a predictor of poorer long-term overall survival. Hypopharyngeal cancers are uncommon and account for 0.4% of all new cancers worldwide [25]. In Taiwan, hypopharyngeal cancers account for 12% of all HNCs and have a 6% increase in the average annual percentage change [5]. Hypopharyngeal cancers are well known to have the worst prognosis of all HNCs, owing to the advanced stage at presentation [26]. Unsurprisingly, in our cohort, patients with hypopharyngeal cancer bleeding had the poorest 1-year survival rate of 13% compared to those ranging from 50–59% in patients with HNC at other sites. Nonetheless, hypopharyngeal cancer also contributed to decreased short-term survival. We speculate that bleeding from this site may induce airway obstruction, cause aspiration of blood, and lead to asphyxiation. Given the silent anatomical location of the hypopharynx, patients have difficulty clearing blood by coughing or vomiting, and recurrent aspiration results in chronic lung inflammation. These bleeding characteristics may exert an additive effect with an associated advanced tumor burden on short-term and long-term survival. The present study also showed that M stage was an independent risk factor for in-hospital mortality in patients with HNC bleeding. Although the presence of distant metastasis is traditionally a prognostic factor for survival in HNC patients, it has not yet been reported in the literature among those presenting with a bleeding event [27].

In addition to cancer-related risk factors, we found that initial presentation of heart rate >110 (beats/min) and inotropic support were independent predictors of poorer long-term survival. HNC patients can manifest with bleeding from minor tumor oozing to episodes of severe hemorrhage. Patients may expire in a short period if they develop hemorrhagic shock. Hemodynamic status is a plausible prognostic factor in patients with massive hemorrhage because hypovolemia and vasoconstriction contribute to tissue hypoperfusion and multiorgan failure [28,29,30,31].

## 5. Limitations

The present study had several limitations. First, due to the retrospective nature, this study did not allow collection of accurate clinical variables, such as past medical history and lifestyle factors. Second, while the present study is the largest study on HNC bleeding patients, the small number of specific cancer subsites limited the applicability of our findings. For example, in our cohort, there were only four laryngeal cancer patients, precluding clear-cut conclusions to be made according to specific cancer subsites. Last, this was a single-country study, and the race and ethnicity in Taiwan are relatively homogenous. To generalize the results of this study, physicians must consider the differences in patients’ individual risk profiles across races.

## 6. Conclusions

HNC bleeding commonly represents the advanced disease stage. In the present study, the in-hospital mortality rate was 16%, and the median survival time was 9.2 months. Inotropic support, hypopharyngeal cancer, and distant metastasis were associated with increased in-hospital mortality, while inotropic support, heart rate >110 (beats/min), and hypopharyngeal cancer were independent predictors of poorer long-term overall survival. Recognition of associated factors aids in the risk stratification of patients with HNC bleeding.

## Figures and Tables

**Table 1:** Characteristics of patients with head and neck cancer bleeding according to survival status.

Variable	Survivors *N* = 105	Nonsurvivors *N* = 20	*p* Value
Age (year)	56.1 ± 11.1	54.9 ± 11.1	0.650
Male	97 (92.4)	17 (85.0)	0.381
Systolic blood pressure (mmHg)	134.7 ± 34.3	133.7 ± 40.8	0.917
Diastolic blood pressure (mmHg)	82.0 ± 17.7	85.4 ± 19.8	0.457
Heart rate > 110 (beats/min)	32 (30.5)	9 (47.4)	0.150
Smoking history	79 (75.2)	18 (90.0)	0.240
Betel nut chewer	69 (65.7)	12 (60.0)	0.624
**Previous medical history**			
Hypertension	30 (28.6)	8 (40.0)	0.309
Diabetes mellitus	18 (17.1)	6 (30.0)	0.216
Coronary artery disease	5 (4.8)	1 (5.0)	1.000
Chronic kidney disease	5 (4.7)	1 (5.0)	1.000
Other malignancy	6 (5.7)	2 (10.0)	0.613
Prior stroke	9 (8.6)	0 (0)	0.352
Liver cirrhosis	10 (9.5)	1 (5.0)	1.000
**Laboratory exam**			
White cell count (10^3^/uL) *n* = 123	11.8 ± 7.9	11.3 ± 5.9	0.784
Hemoglobin (g/dL) *n* = 124	10.8 ± 2.2	10.1 ± 1.8	0.189
Platelet count (10^3^/uL) *n* = 123	274 ± 102	311 ± 159	0.322
INR *n* = 115	1.14 ± 0.10	1.32 ± 0.35	0.048
Creatinine (mg/dL) *n* = 123	0.99 ± 0.94	1.46 ± 1.84	0.278
ALT (U/L) *n* = 102	26.7 ± 18.1	85.0 ± 142	0.111

Count data are expressed as number (percentage) and continuous values are expressed as mean ± SD. INR: international normalized ratio; ALT: alanine aminotransferase.

**Table 2:** Features of cancer and bleeding in patients with head and neck cancer according to survival status.

Variable	Survivors *N* = 105	Non-Survivors *N* = 20	*p* Value
**Cancer site**			0.105
Oral cavity	48 (45.7)	7 (35.0)	
Nasopharynx	17 (16.2)	3 (15.0)	
Oropharynx	22 (21.0)	2 (10.0)	
Hypopharynx	14 (13.3)	8 (40.0)	
Larynx	4 (3.8)	0 (0)	
**T stage**			0.294
T ≤ 2	33 (31.4)	3 (15.0)	
T > 2	68 (64.8)	16 (80.0)	
Unknown	4 (3.8)	1 (5.0)	
**N stage**			0.656
N0	34 (32.4)	6 (30.0)	
N+	64 (61.0)	14 (70.0)	
Unknown	7 (6.7)	0 (0)	
**M stage**			0.157
M0	95 (90.5)	16 (80.0)	
M1	4 (3.8)	3 (15.0)	
Unknown	6 (5.7)	1 (5.0)	
**Pathology type**			0.745
Squamous cell carcinoma			
Keratinizing carcinoma	96 (91.4)	18 (90.0)	
Non-keratinizing carcinoma	7 (6.7)	2 (10.0)	
Sarcomatoid carcinoma	1 (1.0)	0 (0)	
Adenocarcinoma	1 (1.0)	0 (0)	
**Initial cancer treatment**			
Surgical resection	51 (48.6)	8 (40.0)	0.482
Chemoradiation	83 (79.0)	17 (85.0)	0.762
Neck dissection	45 (42.9)	7 (35.0)	0.514
Flap reconstruction	36 (34.3)	7 (35.0)	0.951
**Local recurrence**	49 (46.7)	9 (45.0)	0.891
**Bleeding cause**			0.268
Tumor related	78 (74.3)	18 (90.0)	
Pseudoaneurysm	19 (18.1)	2 (10.0)	
Post-operative complication	8 (7.6)	0 (0)	
**Bleeding type**			0.120
Self-limited	40 (38.1)	4 (20.0)	
Active bleeding	65 (61.9)	16 (80.0)	
**Emergent CTA**	49 (46.7)	13 (65.0)	0.133
**Bleeding treatment**			0.952
Supportive care	77 (73.3)	16 (80.0)	
Embolization	19 (18.1)	4 (20)	
Covered stent	5 (4.8)	0 (0)	
Surgical ligation	3 (2.9)	0 (0)	
Primary repair	1 (1.0)	0 (0)	
**Inotropic support**	3 (2.9)	4 (20.0)	0.013
**Blood transfusion**	54 (51.4)	11 (55.0)	0.770

Count data are expressed as number (percentage) and continuous values are expressed as mean ± SD. CTA: computed tomography angiography.

**Table 3:** Univariate and multivariate analyses of risk factors for in-hospital mortality with logistic regression.

	Univariate		Multivariate	
	OR (95%CI)	*p* Value	OR (95%CI)	*p* Value
Age	0.99 (0.95, 1.03)	0.647		
Male	0.47 (0.11, 1.94)	0.295		
Heart rate > 110 (beats/min)	2.05 (0.76, 5.53)	0.155		
Inotropic support	8.50 (1.74, 41.56)	0.008	10.41 (1.81, 59.84)	0.009 *
Hypertension	1.67 (0.62, 4.48)	0.312		
Diabetes mellitus	2.07 (0.70, 6.12)	0.187		
Hypopharyngeal cancer	4.33 (1.51, 12.47)	0.007	4.32 (1.29, 14.46)	0.018 *
Surgical resection	0.71 (0.27, 1.87)	0.483		
Chemoradiation	1.50 (0.40, 5.59)	0.544		
Neck dissection	0.72 (0.27, 1.95)	0.515		
Flap reconstruction	1.03 (0.38, 2.82)	0.951		
T stage	1.84 (1.01, 3.37)	0.050	1.36 (0.70, 2.64)	0.368
N stage	1.05 (0.66, 1.68)	0.827		
M stage	4.45 (0.91, 21.79)	0.065	5.90 (1.07, 32.70)	0.042 *
Local recurrence	0.94 (0.36, 2.44)	0.891		

OR: odds ratio; 95% CI: 95% confidence interval. * *p* value < 0.05.

**Table 4:** Univariate and multivariate analyses of predictors for poorer long-term survival with Cox proportional hazards model.

	Univariate		Multivariate	
	HR (95%CI)	*p* Value	HR (95%CI)	*p* Value
Age	0.99 (0.97, 1.01)	0.225		
Male	0.63 (0.30, 1.31)	0.215		
Heart rate > 110 (beats/min)	1.95 (1.18, 3.23)	0.010	2.02 (1.16, 3.51)	0.013 *
Inotropic support	5.71 (2.40, 13.57)	<0.001	3.25 (1.20, 8.82)	0.021 *
Hypertension	0.76 (0.44, 1.30)	0.309		
Diabetes mellitus	1.02 (0.56, 1.07)	0.943		
Hypopharyngeal cancer	2.79 (1.60, 4.85)	<0.001	2.22 (1.21, 4.06)	0.010 *
Surgical resection	0.57 (0.35, 0.92)	0.023	0.74 (0.42, 1.31)	0.301
Chemoradiation	2.55 (1.21, 5.37)	0.014	2.10 (0.89, 4.94)	0.094
Neck dissection	0.76 (0.47, 1.23)	0.260		
Flap reconstruction	0.88 (0.53, 1.45)	0.603		
T stage	1.40 (1.10, 1.78)	0.006	1.26 (0.96, 1.65)	0.092
N stage	1.33 (1.06, 1.68)	0.015	1.19 (0.90, 1.58)	0.213
M stage	2.13 (0.76, 5.96)	0.149		
Local recurrence	1.95 (1.20, 3.19)	0.007	1.76 (0.99, 3.12)	0.051

HR: hazard ratio; 95% CI: 95% confidence interval. * *p* value < 0.05.

## Data Availability

Not applicable.
